# Across-line SNP association study for (innate) immune and behavioral traits in laying hens

**DOI:** 10.1186/1753-6561-5-S4-S18

**Published:** 2011-06-03

**Authors:** Jan J van der Poel, Filippo Biscarini, Bas T Rodenburg, Johan AM van Arendonk, Henk K Parmentier, Annemieke P Jungerius, Henk Bovenhuis

**Affiliations:** 1 Animal Breeding and Genomics Centre, Wageningen University, PO Box 338, 6700 AH Wageningen, The Netherlands; 2 Adaptation Physiology Group, Wageningen University, PO Box 338, 6700 AH Wageningen, The Netherlands; 3 Hendrix Genetics, Research & Technology Centre, Spoorstraat 69, 5830 AC Boxmeer, The Netherlands

## Abstract

**Background:**

An association study between single nucleotide polymorphism markers (SNP) and (innate and adaptive) immune parameters but also feather condition score on the back, rump and belly of laying hens was performed. The immune parameters measured in blood samples were natural and acquired antibody titers and complement activity. Feather condition score as a measure of feather damage was determined, this parameter is closely related to feather pecking behavior in hens housed in groups.

The aim of the study was to detect associations between genetic markers and immune parameters and feather condition score across nine lines of laying hens, focusing on the feather peckers as well as on the victims of feather pecking.

**Methods:**

A novel approach based on across-line analysis and testing of the SNP-by-line interaction was performed.

**Results:**

In total 59 significant associations between SNP and immune traits were detected. Previously identified QTL were confirmed and new associations of genes regulating immune function identified. The IL17A gene (chromosome 3) influences natural and acquired antibody titers and activation of classical and alternative complement pathways. The major histocompatibility complex on chromosome 16 showed significant association with natural and acquired antibody titers and classical complement activity. The IL12B and IRF1 genes on chromosome 13 were associated with natural antibody titers.

The direct effect of the genotype of an individual on its feather condition and the associative effect of the genotype of the cage mates on the individual’s feather condition were analyzed. The direct genetic effect can be described as the susceptibility to be pecked at, and the associative genetic effect as the propensity to perform feather pecking. Eleven significant associations were detected for the direct effect, and 81 for the associative effect. The serotonin receptor 2C (HTR2C) on chromosome 4 was highlighted in both analyses.

**Conclusions:**

Our results confirmed previously identified QTL and identified new associations of genes regulating immune function. The results for feather condition score supports existing evidence of involvement of the serotonergic system in feather pecking in laying hens. Immune regulatory genes were found to be associated to feather condition score, revealing relationships between the immune system and behavior.

## Background

Immunity is composed of innate and adaptive compartments and parameters can be measured e.g. as antibody titers and the activity of the complement system in the blood. Natural antibodies (NAbs) and the complement system define innate humoral immunity and constitute a substantial part of the basic capacity of an organism to respond to pathogens [1]. Acquired antibodies neutralize pathogens upon exposure of an individual and are part of the adaptive humoral immunity. NAbs and complement titers have been associated with survival in laying hens [2]. Identification of genes regulating these immunological parameters is of interest, as this information could be used to select for animals with superior immune response.

Feather pecking (FP) is a serious behavioral disorder of laying hens. FP is a multi-factorial problem caused by both genetic and environmental factors. Line differences in FP have been documented [3,4]. Feather pecking is heritable [5,6] and chromosomal regions involved in feather pecking have been identified [7,8]. In relation to FP and cannibalism social interactions are important and it was shown that the associative effect due to the genotypes of group mates can contribute substantially to the total heritable variation [9,10]. Because measurement of FP is labor intensive, instead feather condition scores was used to assess plumage condition because of the relation to feather pecking activity [11].

## Methods

A population of 583 hens (for immune parameters) and 660 hens (for feather score) was genotyped for 1022 SNP and used for the association study. The animals came from 4 Rhode Island Red and 5 White Leghorn pure lines. On average 65 hens per line were tested for immunological parameters and 73 hens per line for feather condition score. Phenotypes for immune parameters were described in detail by Star et al. [2]. Feather condition score was previously described by Uitdehaag et al. [4].

The approach based on the analysis of multiple lines and tests of SNP-by-line interaction in a population of laying hens measured for several immune traits and feather condition score is described in detail by Biscarini et al. [12,13]. Among lines, linkage disequilibrium is conserved at shorter distances than in individual lines; therefore, SNP significantly associated with traits across lines are expected to be closer to functional mutations. In the analysis, the SNP that had a significant across-line effect but did not show significant SNP-by-line interaction were identified to test whether the association was consistent in the individual lines [14].

## Results

### Immune traits

The SNP rs14050302 located in the IL12B (interleukin 12B) gene on chromosome 13 was linked to the production of NAbs (Table 1) [12]. Two SNP within 0.1 cM of this locus (rs15677371 and rs15677377) were also associated with NAb levels. These results emphasize a role for IL12B in regulating the immune response. The association of the IRF1 gene, being in close proximity to IL4, suggests that also IL4 may be involved in the regulation of NAb levels. The results suggest an important yet unexpected involvement of the MHC region and IL-12B, IL-17A and IL10 cytokines in the regulation of NAbs. This is the first indication in either poultry or mammals that NAbs levels are regulated by these genes.

**Table 1:** SNP significantly associated with immune traits

						Complement activity	
**chromosome**	**SNP^1^**	**Kbps**	**cM^2^**	**Natural Abs**	**Acquired Abs**	**classical**	**alternative**

chr 3	rs13526054 (*IL17A*)^3^	110370	275.9	+	-	+	+
	rs15458146 (*IL17A*)^3^	110371	275.9	+	+	-	-

chr 13	rs15677371	7927	19.8	+	-	-	-
	rs15677377	7927	19.8	+	-	-	-
	rs14050302(*IL12B*)^3^	7917	19.8	+	-	-	-
	rs14064896 (*IRF1*)^3^	17452	43.6	+	-	-	+
	rs14064900 (*IRF1*)^3^	17452	43.6	-	+	+	-

chr16	snp.gga16Tapasin. 180144exon1TC (*TAPBP*)^3^	65	0.2	-	+	+	-
	rs15788216 (*MHC*/*BLB1*)^3^	70	0.2	+	+	-	-

chr 26	rs14298900 (*IL10*)^3^	2375	5.9	+	-	+	-

1. SNP as present in dbSNP at NCBI (http://www.ncbi.nlm.nih.gov).

2. 1 cM = 4 · 105 bps.

3. IL17A, gene encoding interleukin 17A; HTR2C, gene encoding the serotonin receptor 2C; IL12B, gene encoding interleukin 12B; IRF1, gene encoding interferon regulatory transcription factor; MHC, gene encoding major histocompatibility complex; BLB1, gene encoding class II beta chain 1; TAPBP, gene encoding TAP binding protein; IL10, gene encoding interleukin 10.

### Behavioral traits

In the analysis of direct genetic effects, the SNP rs15385785 located in the MAOA gene on chromosome 1 and rs13640917 in the serotonin receptor gene 2C (HTR2C, chromosome 4) showed significant associations with feather scores[13]. In total 11 SNP showed significant associations for the direct genetic effect. In the associative analysis 57 SNP showed significant associations (p <0.05). Two SNP proved to be significantly associated with feather condition score both in the direct and associative model: SNPs rs13640917 on chromosome 4 (HTR2C) and rs14999300 on chromosome 13 (IL9). Several genes for transporter molecules, which could play a role in behavioral disorders, were detected. In addition the IL4 gene and a chemokine receptor (CCL4) showed a significant effect on feather score. (For details see [13])

## Discussion

In this study, we presented an original approach to detect SNP associated with immune and behavioral traits. The method is based on the simultaneous analysis of multiple lines using a multiple-step procedure. In the across-line analysis, the SNP detected were expected to be close the QTL for a trait because of the reduced extent of LD conserved across lines. Building on the work by Saccone et al. [14], we tested for the SNP-by-line interaction to ensure consistency of the association across lines. A possible source of false positive associations because of population stratification was avoided by including a line effect in the model. Consequently, SNP explaining part of the between-line variation could not be detected in this approach.

The across line analysis was successful in detecting significant associations of SNP for innate immune as well as behavioral traits. The study shows that genetic variants of IL17, IL12, IL10 and IRF1 (located in the proximity of IL4) and the major histo-compatibility complex encoded genes (BLB and TAPBP) affect NAb and/or acquired antibody levels. In addition complement activity of classical or alternative pathway was affected.

The analysis for behavioral traits emphasized the role of the serotonergic system in pecking behavior, the HTR2C gene showed significant association in the direct as well as the associative model. The fact that more significant associations were found in the associative model (n=57), as opposed to the direct model (n=11), is in line with the observation that heritability estimates are higher for performing FP than receiving FP [5,6]. Associative effects contribute substantially to the total heritable variation, and have been shown to be larger in magnitude than direct genetic effects [9,10].

The relations between behavior and immunity as demonstrated in our analyses by the significant associations with immune regulatory genes, is in line with previous studies by our group [15]. Low levels of innate immunity, either cellular or humoral, might be related to disease susceptibility, whereas high levels might be related to disease resistance [16]. This reinforces the notion that Nabs might be important for the maintenance of homeostasis and disease resistance [17].

## Conclusions

The study confirmed previously identified QTL and identified new associations of genes regulating immune function. The results for feather condition score supports existing evidence of involvement of the serotonergic system in feather pecking in laying hens. Immune regulatory genes were found to be associated to feather condition score, revealing relationships between the immune system and behavior.

## Abbreviations

FP, feather pecking; NAb, natural antibody; SNP, single nucleotide polymorphism.

## Competing interests

The authors declare that they have no competing interests.

## Authors' contributions

FB and HB performed the association studies, HP and JvdP were responsible for the immune traits, BR was responsible for the behavioral traits, AJ and JvdP designed and performed SNP genotyping. All authors contributed to the design of the study and revision of the paper.
